# The addition of a sagittal image fusion improves the prostate cancer detection in a sensor-based MRI /ultrasound fusion guided targeted biopsy

**DOI:** 10.1186/s12894-016-0196-9

**Published:** 2017-01-13

**Authors:** Karsten Günzel, Hannes Cash, John Buckendahl, Maximilian Königbauer, Patrick Asbach, Matthias Haas, Jörg Neymeyer, Stefan Hinz, Kurt Miller, Carsten Kempkensteffen

**Affiliations:** 1 Department of Urology, Charité — University Medicine Berlin, Hindenburgdamm 30, 12203 Berlin, Germany; 2 Departement of Radiology, Charité — University Medicine Berlin, Hindenburgdamm 30, 12203 Berlin, Germany

**Keywords:** Multiparametric magnetic resonance imaging, Targeted biopsy, Prostate cancer detection, MRI/US fusion biopsy

## Abstract

**Background:**

To explore the diagnostic benefit of an additional image fusion of the sagittal plane in addition to the standard axial image fusion, using a sensor-based MRI/US fusion platform.

**Methods:**

During July 2013 and September 2015, 251 patients with at least one suspicious lesion on mpMRI (rated by PI-RADS) were included into the analysis. All patients underwent MRI/US targeted biopsy (TB) in combination with a 10 core systematic prostate biopsy (SB). All biopsies were performed on a sensor-based fusion system. Group A included 162 men who received TB by an axial MRI/US image fusion. Group B comprised 89 men in whom the TB was performed with an additional sagittal image fusion.

**Results:**

The median age in group A was 67 years (IQR 61–72) and in group B 68 years (IQR 60–71). The median PSA level in group A was 8.10 ng/ml (IQR 6.05–14) and in group B 8.59 ng/ml (IQR 5.65–12.32). In group A the proportion of patients with a suspicious digital rectal examination (DRE) (14 vs. 29%, *p* = 0.007) and the proportion of primary biopsies (33 vs 46%, *p* = 0.046) were significantly lower. The rate of PI-RADS 3 lesions were overrepresented in group A compared to group B (19 vs. 9%; *p* = 0.044). Classified according to PI-RADS 3, 4 and 5, the detection rates of TB were 42, 48, 75% in group A and 25, 74, 90% in group B. The rate of PCa with a Gleason score ≥7 missed by TB was 33% (18 cases) in group A and 9% (5 cases) in group B; p-value 0.072. An explorative multivariate binary logistic regression analysis revealed that PI-RADS, a suspicious DRE and performing an additional sagittal image fusion were significant predictors for PCa detection in TB. 9 PCa were only detected by TB with sagittal fusion (sTB) and sTB identified 10 additional clinically significant PCa (Gleason ≥7).

**Conclusion:**

Performing an additional sagittal image fusion besides the standard axial fusion appears to improve the accuracy of the sensor-based MRI/US fusion platform.

**Electronic supplementary material:**

The online version of this article (doi:10.1186/s12894-016-0196-9) contains supplementary material, which is available to authorized users.

## Background

Prostate cancer (PCa) is the most common malignancy of men and the only tumour, which is diagnosed according to the guidelines by untargeted systematic biopsies of the entire organ [1, 2]. Because prostate cancer is often not visualized in conventional transrectal ultrasound, there is a risk to miss clinically significant PCa (Gleason ≥7) with a random systematic transrectal prostate biopsy (SB) [3, 4]. Due to a high soft-tissue contrast, a high resolution (T2-weighted anatomical sequences) and the registration of functional parameters (diffusion-weighted and dynamic contrast-enhanced sequences (DWI and DCE), MR spectroscopy imaging) a multiparametric magnetic resonance imaging (mpMRI) of the prostate provides a high sensitivity, specificity and negative predictive value in the detection and localization of clinically significant prostate cancers [5, 6]. For standardization of evaluation of the mpMRI the “European Society of Urogenital Radiology” (ESUR) established the “Prostate Imaging Reporting and Data System” (PI-RADS), which introduced a 5-point Likert scale for each region (peripheral and central glandular sections) with corresponding scores for each sequence (T2, DWI, DCE, and MR-Spectroscopy) [7, 8]. The correlation of the level of PI-RADS with the overall detection rate of PCa and the detection of significant PCa has been demonstrated in various studies [9–13]. The increasing utilization of mpMRI of the prostate and the consecutive MRI/ultrasound fusion guided targeted biopsy (TB) resulted in an improved detection of PCa compared to SB, the current standard of care [14–17]. A difficulty is the exact fusion of mpMRI with transrectal ultrasound for TB. Various possibilities of MRI/ultrasound (MRI/US) image fusion, such as cognitive fusion, sensor-based fusion or organ-based fusion are available to perform TB. Despite the technological progress of different fusion platforms, several studies have shown that clinically significant PCa can still be overlooked by TB [17–20]. For the sensor-bases TB we previously analyzed the possible pitfalls of TB, such as reader variability for mpMRI, an imprecise targeting of the suspicious lesion [21]. Traditionally sensor-based fusion of the MRI image and the real-time ultrasound image is performed by the operator in the axial plane according to anatomical landmarks (i.e. prostatic apex, periprostatic vessels, BPH nodes etc.). In order to further improve the targeting accuracy and reduce a possible image fusion error, this study evaluated the use of an additional image fusion in the sagittal plane according to a 3-point technique. In a cohort of 791 men, who underwent a MRI/US fusion biopsy with an organ-based fusion system, Hong et al. demonstrated that the combination of axial and sagittal approaches detected more clinically significant PCa [22]. For sensor-based fusion platforms there is currently no data on the effect of an added sagittal image fusion.

## Methods

### Study population

In the period of July 2013 to September 2015, 251 patients, who showed at least one suspicious lesion on mpMRI (PI-RADS ≥3) and underwent a consecutive TB in combination with a 10-core systematic prostate biopsy (SB), were consecutively included into the retrospective analysis. The indication for a mpMRI has largely been provided by attending outpatient urologists. Parts of the cohort were analysed in a previous study [13]. All patients were recorded regardless to the number of prior prostate biopsies. The data collection was based on the patients medical history, clinical findings and the physical patient files. Patient data was prospectively collected in a START conform database [23]. The analysis in regard to the axial and sagittal image fusion was performed retrospectively. All patients signed an informed consent for the intervention, data acquisition and data evaluation. The study was performed according to the declaration of Helsinki and the analysis was approved by the Institutional Review Board of the Charité University Medicine Berlin.

### Multiparametric magnetic resonance imaging

A 3-Tesla mpMRI (Magnetom Skyra, Siemens Medical Systems, Erlangen, Germany) without an endorectal coil was performed for all patients before prostate biopsy. The MRI protocol contained high spatial resolution T2-weighted turbo spin-echo sequences in axial, sagittal and coronal orientation, axial turbo spin-echo T1 weighted images, axial diffusion weighted images (b-values 0.400 and 800 s/mm^2^) and dynamic contrast-enhanced sequences. The evaluation of the mpMRI was performed by experienced radiologists according to the guidelines of the European Society of Urogenital Radiology (ESUR) [8]. From a PI-RADS score of 3, the indication for MRI/US fusion biopsy was made.

### MRI-ultrasound fusion prostate biopsy and systematic biopsy

The prostate biopsies were performed under antibiotic prophylaxis with a fluoroquinolone according to the EAU guidelines [2], with a high-end ultrasound device HiVision Preirus (Hitachi Medical Systems, Tokyo, Japan) and an endocavity endfire probe (EUP V53W, Hitachi Medical Systems, Tokyo, Japan). All biopsies were taken in lithotomy position. At first TB were performed. T2 and DWI sequences of the axial planes in mpMRI were imported to the ultrasonic device. After that, the suspicious lesions were marked in axial orientation of the mpMRI sequences by the urologist experienced with mpMRI. The MRI/US image fusion was performed using sensor-based registration. The movement of the probe with an attached tracker was detected in a low magnetic field (0.1 Tesla), which was generated by a mini bird receiver. Until December 2014 only axial MRI/US image fusion was performed. For this purpose, the same plane in ultrasound and MRI was identified according to anatomical landmarks (prostatic apex, periprostatic vessels, BPH nodes, intraprostatic cysts) Depending on the anatomical conditions, the angle of axial plane in the MRI image was corrected to match the angulation of the ultrasound probe and image. The previously marked suspect lesions were transferred to the real-time ultrasound image by the platform’s software, followed by a 2–5 targeted biopies in an axial orientation. After an analysis of possible reasons for targeted biopsy failure, as of December 2014 the targeted biopsies were performed after MRI/US image fusion in both the axial and sagittal plane [21]. The total number of targeted biopsies remained unchanged. For the sagittal image fusion, a T2-weighted sequence in sagittal orientation was used to mark the bladder neck, the apex of the prostate and the seminal vesicle angle. Based on these marks the MRI and the ultrasound image were fused by the software. Thereafter, TB was carried out in a sagittal orientation of the MRI and ultrasound image. For sampling, we used a long biopsy needle (18 g × 25 cm, Bard Magnum biopsy instrument, Tempe, United States). After performing TB, local anaesthesia with a bupivacaine was injected at the dorsal prostatic capsule and a 10-core SB was conducted without changing the examiner. The SB scheme included cores from: left/right apex, left/right lateral mid gland, left/right base, left/right ventral and left/right para-urethral. All tissue-samples were documented by their extraction location and shipped separately for histopathological evaluation by our experienced pathologists.

### Group distribution

**Fig. 1:**
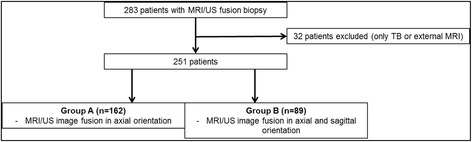
Patient inclusion and group distribution. Group A included patients between July 2013 and December 2014 where an axial targeted biopsy was the standard Group B included patients between December 2014 and September 2015 where and axial and sagittal targeted biopsy was performed without increasing the total number of targeted biopsy cores

Group A included all patients who have received an MRI/US image fusion only in the axial plane. Group B, are included all patients who have received MRI/US image fusion in the axial and sagittal plane. Figure 1 shows a flow chart for the patient inclusion and the group distribution.

### Statistical analysis

PASW Version 22 (SPSS Inc. 1998–2010, Chicago, Illinois 60606, USA) was used for statistical analyses. Categorical data were presented by absolute and relative frequencies. Continuous variables were measured by means and standard deviation when normal distributed or by medians and quartiles. Continuous variables were evaluated with the Kolmogorov-Smirnov-test for normal distribution. We used chi-square test, Student’s t-test and Mann-Whitney U test to calculate statistical differences between numerical and categorical variables. Univariate and multivariate binary regression analysis were performed to evaluate significant parameters in the descriptive analysis as predictors for PCa detection. A p-value of *p* <0.05 was considered statistical significant.

## Results

Demographic data, clinical characteristics and MRI findings are presented in Table 1 divided in group A (patients without additional sagittal image fusion) and group B (patients with additional sagittal image fusion). The median age in group A was 67 years (IQR 61–72) and in group B 68 years (IQR 60–71). Both groups showed statistically similar prostate volumes (48 vs. 50 ml). The median PSA level in group A was 8.10 ng/ml (IQR 6.05–14.00) and in group B 8.59 ng/ml (IQR 5.65–12.32). The proportion of patients with a suspicious digital rectal examination (DRE) (14 vs. 29%, *p* = 0.007) and the proportion of patients with primary biopsies (33 vs 46%, *p* = 0.041) were significantly lower in group A. The rate of PI-RADS 3 lesions were overrepresented in group A (19 vs. 9%; *p* = 0.044). With 43% compared to 30% in group A PI-RADS 5 lesions were more frequently represented in group B (*p* = 0.051). No significant differences were observed for lesion positions, number of suspicious lesions in mpMRI and lesion sizes. The mean number of cores taken per patient and the mean number of TB per patient were significant higher in Group A (13.7 vs. 13.2 and 3.8 vs. 3.4; *p* = 0.009 and 0.031). The analysis showed a significant higher overall cancer detection rate (CDR) (85 vs. 72%; *p* = 0.019) and a significant higher detection rate in TB (76 vs. 55%; *p* = 0.001) in group B, please see Table 2. Furthermore there was a significant lower number of patients diagnosed with a clinically significant PCa in group A (61 vs. 78%; *p* = 0.025). Classified according to PI-RADS 3, 4 and 5, the detection rates of TB were 42, 48, 75% in group A and 25, 74, 90% in group B. The rate of PCa with a Gleason score ≥7 missed by TB was **33%** (18 cases) in group A and **9%** (5 cases) in group B (*p* = 0.072). The overall cancer detection rates and the PI-RADS based analyses for SB and TB for men without suspicious DRE and prior negative biopsy are shown in Additional file 1 Table S1 and Additional file 2 Table S2. An explorative multivariate binary logistic regression analysis revealed that PI-RADS, a suspicious DRE and performing an additional sagittal image fusion were significant predictors for PCa detection in TB, ﻿please see Table 3﻿. Table 4 shows the comparison of the biopsy results of the axial (aTB) and sagittal (sTB) MRI/US fusion biopsy. Depending on PI-RADS, lesion diameter and lesion localization, PCa detection rates of aTB and sTB were statistically equivalent except a higher detection of PCa by aTB for PI-RADS 4 lesions (70 vs. 44%, *p* = 0.007). Furthermore, Table 4 shows the additional detection of PCa in total and of PCa with a Gleason score ≥7 due sTB depending on PI-RADS, lesion diameter and lesion localization. Overall, nine PCas were only detected by sTB and sTB identified 10 additional clinically significant PCa (Gleason ≥7).

**Table 1:** Patient demographics and magnetic resonance imaging findings (*n* = 251)

	Group A (*n* = 162)	Group B (*N* = 89)	*p*-value
Median (IQR) age, years	67 (61–72)	68 (60–71)	0.846
Median (IQR) PSA. ng/ml	8.10 (6.05–14.00)	8.59 (5.65–12.32)	0.997
Median IQR) f/t PSA-ratio,%	12 (9–17)	13 (9–19)	0.309
Median (IQR) prostate volume, ml	48 (35–60)	50 (37–70)	0.087
Suspicous DRE, n (%)	23 (14%)	26 (29%)	0.007
No. of prior biopsies, n (%)			
Primary biopsy	53 (33%)	41 (46%)	0.041
1	59 (36%)	28 (32%)	0.489
2	33 (20%)	13 (15%)	0.308
≥3	17 (10%)	7 (8%)	0.655
Localization of lesions with maximum PI-RADS on mpMRI, n (%)			
Apex	72 (44%)	35 (39%)	0.505
Midgland	43 (27%)	32 (36%)	0.149
Base	47 (29%)	22 (25%)	0.555
Anterior	45 (28%)	28 (32%)	0.563
Median (IQR) no. of lesions per patient	1 (1–2)	1 (1–2)	0.451
Maximum diameter of lesions (mm)	14 (10–17)	13 (10–18)	0.885
Mean (SD) of cores taken per patient	13.7 (±1.6)	13.2 (±1.2)	0.009
Mean (SD) TBs per patient	3.8 (±1.5)	3.4 (±0.9)	0.031
Maximum mpMRI Score, n (%)^a^			
PI-RADS 3	31 (19%)	8 (9%)	0.044
PI-RADS 4	83 (51%)	43 (48%)	0.693
PI-RADS 5	48 (30%)	38 (43%)	0.051

*PSA* prostate-specific antigen, *IQR* interquartile range, *SD* standard deviation, *DRE* digital rectal examination, *mpMRI* multiparametric magnetic resonance imaging, *PI-RADS* Prostate Imaging Reporting and Data System, *TB* targeted biopsy

^a^For patients with multiple lesions the highest PI-RADS score is stated

**Table 2:** Cancer Detection Rate and Gleason pattern

	Group A (*n* = 162)	Group B (*N* = 89)	*p*-value
Overall CDR	117 (72%)	76 (85%)	0.019
SB	108 (67%)	68 (76%)	0.115
TB	89 (55%)	68 (76%)	0.001
*PI-RADS 3 (n = 39)*			
Overall CDR	17 (55%)	5 (63%)	>0.999
SB	15 (48%)	3 (38%)	0.702
TB	13 (42%)	2 (25%)	0.450
*PI-RADS 4 (n = 126)*			
Overall CDR	55 (66%)	36 (84%)	0.058
SB	48 (58%)	33 (77%)	0.049
TB	40 (48%)	32 (74%)	0.007
*PI-RADS 5 (n = 86)*			
Overall CDR	45 (94%)	35 (92%)	>0.999
SB	45 (94%)	32 (84%)	0.175
TB	36 (75%)	34 (90%)	0.102
Detected GS ≥7 in TB	54 (61%)	53 (78%)	0.025
Missed PCa (GS ≥7) in TB	18 (33%*)	5 (9%*)	0.072

CDR = Cancer Dection Rate; GS = Gleason Score; SB = Random Biopsy; TB = Target biopsy; * % of GS ≥7 detected by TB

**Table 3:** Predictors for prostate cancer detection in the Targeted Biopsy

	Univariate analysis		Multivariate analysis	
	OR	*p*-value	OR	*p*-value
Suspicious DRE	4.539	<0.001	2.777	0.024
Primary biopsy	1.175	0.553		
PI-RADS	2.712	<0.001	2.240	<0.001
Sagittal image fusion	2.656	0.001	2.105	0.017

**Table 4:** Cancer Detection Rate of the Targeted Biopsy in relation to an axial and sagittal image fusion

Group B n = 89	Overall CDR in TB (sTB + aTB)	CDR in aTB	CDR in sTB	Additional PCa detected only by sTB*	Additional GS ≥7 in sTB^#^
Overall	68 (76%)	59 (66%)	50 (56%)	9 (13%)	10 (19%)
*PI-RADS*					
3 (*n* = 8)	2 (25%)	2 (25%)	2 (25%)	0	0
4 (*n* = 43)	32 (74%)	30 (70%)	19 (44%)	2 (5%)	4 (9%)
5 (*n* = 38)	34 (90%)	27 (71%)	29 (76%)	7 (18%)	6 (16%)
*Maximum diameter of lesion*					
1–10 (*n* = 27)	18 (67%)	15 (56%)	10 (37%)	3 (11%)	3 (11%)
11–20 (*n* = 50)	38 (76%)	33 (66%)	29 (58%)	5 (10%)	5 (10%)
>20 (*n* = 12)	12 (100%)	11 (92%)	11 (92%)	1 (8%)	2 (17%)
*Localization of lesion*					
Apex (*n* = 35)	26 (74%)	23 (66%)	19 (54%)	3 (9%)	3 (9%)
Midgland (*n* = 32)	23 (72%)	18 (56%)	15 (47%)	5 (16%)	5 (16%)
Base (*n* = 22)	19 (86%)	18 (82%)	16 (73%)	1 (5%)	2 (9%)
Anterior (*n* = 28)	26 (93%)	22 (79%)	22 (79%)	4 (14%)	5 (18%)

aTB = axial fusion TB; sTB = sagittal fusion TB; * compared to overall CDR or TB (aTB + sTB);

^#^Either detection of GS ≥7 only by sTB or Gleason upgrade in the sTB biopsy core compared to the aTB core; percentage of GS ≥7 detected by TB (aTB + sTB) *n* = 53

## Discussion

Since the introduction of MRI/US fusion biopsy of the prostate, several studies have demonstrated an improvement in prostate cancer detection rates as well as the identification of clinically relevant tumours [14, 15, 24–26]. Due to this increasing value of MRI/US fusion biopsy for primary diagnosis and monitoring of prostate cancer various fusion systems have been established. A variety fusion systems (UroNav, BiopSee, Urostation, Artemis, HiVisonPreirus, etc.) have been reported in the current literature [13, 14, 22, 24, 26, 27]. Uniform treatment regimens for the implementation of MRI/US fusion biopsies do not exist. In a large patient cohort Hong et al. demonstrated for organ based MRI/US fusion biopsies that the combination of sagittal and axial biopsy approaches identified additional clinically significant prostate cancers [22]. It can be assumed that the correctness of the image fusion of MRI and transrectal ultrasound has an important influence on the accuracy of targeted sampling. Our study showed a significant increase in prostate cancer detection rate of TB by 55% in the group without sagittal fusion to 76% in the group with additional sagittal fusion and the improvement remained even when men with a positive DRE and a primary biopsy were excluded. In addition, the proportion of detected clinically significant PCa (Gleason-score ≥7) increased from 61% in group A to 78% in group B. The sole analysis of the detection rates of axial TB results in an increase of 56 to 66% in group B. The observed increase in detection rates might be related to various factors. In the sensor-based MRI/US image fusion, the same layers in the T2-weighted MRI sequence and the transrectal ultrasound image in axial or sagittal orientation are fused. Identifying the same layers in MRI and US are the basis of fusion accuracy. Angular deviations of the display plane in transrectal ultrasound and MRI lead to inaccuracies of image fusion. In our study, the angle correction for axial image fusion was carried out manually by the urologist. In the sagittal image fusion, the angular offset is software-based by three identical points, which are simultaneously marked in MRI and ultrasound in two different layers. Gaziev et al. showed an increase in the detection rate of prostate cancer by performing perineal MRI/US fusion biopsies of the prostate with increasing experience of the examiner [28]. It is tempting to speculate, that in our study the learning curve of the examiner has likewise lead to an increase in the detection rate in the TB after implementation of additional sagittal image fusion. Another important factor influencing the detection rate of TB is the PI-RADS score [12, 13]. Our study cohort showed a significant decrease of percentage of PI-RADS 3 lesions and a non-significant increase in the proportion of PI-RADS 5 lesions in the patient group with additional sagittal fusion. Also in the univariate and multivariate regression analysis the level of PI-RADS was identified as a significant predictor for PCa detection. This may have occurred to an increased PCa detection in group B, but the sagittal image fusion remained an independent predictor for cancer detection by TB. A suspicious digital rectal examination as described by Radtke et al. and Potter et al. presents a further risk factor for the detection of PCa in TB and SB [18, 29]. Similar to the previously published studies, our univariate and multivariate regression analyses of the whole cohort revealed a significant correlation of a suspicious DRE with PCa detection rate. The higher TB detection rate in group B, that included more men with a suspicious DRE, may therefore have been influenced, but the higher detection rate in group B compared to group A persisted when the analysis excluded men with a suspicious DRE. In our regression analysis, the proportion of biopsy naive men was not a significant predictor for PCa detection, although two large studies showed an influence on cancer detection [12, 22]. Therefore, the significantly higher proportion of primary biopsies in group B may have affected the detection rate of the TB. Again, the improved detection rate in group B remained when men with a positive DRE and a primary biopsy were excluded from the analysis.

The additional implementation of the sagittal image fusion resulted in an increase in the detection rate of 10% for TB. By sagittal fusion, nine (13%) additional prostate cancers were detected and ten (19%) additional clinically significant tumors were identified. The improvement of the axial TB, when adding a sagittal TB was independent of the lesion size or localization of the lesions. Moreover, performing a sagittal image fusion was a significant predictor in univariate and multivariate regression analysis for the detection of prostate cancer in the TB. In the group of patients with sagittal fusion, the proportion of overlooked clinically significant tumors by TB dropped to 9% compared to 33% in the group of patients without sagittal fusion. The reduced rate of missed PCa after the introduction of the sagittal image fusion was not accompanied with an increase of the number of targeted biopsies.

Adding the sagittal image fusion when performing TB on a sensor-based platform may reduce the targeting error that may be inevitable in some cases [21]. In our institution we have therefore established the additional sagittal image fusion firmly in our MRI/US fusion biopsy protocol.

Because of the retrospective study design the investigation has several limitations. Unconsidered confounders may have also influenced the study results, e.g. a selection bias of patients by referring outpatient urologist. The inhomogeneity of the two study groups in terms of baseline characteristics may have affected the study results. To ensure the data consistency, we performed logistic regression analyses for the evaluation of predictors of PCa detection by targeted biopsy. In order to clearly demonstrate the impact of an additional sagittal image fusion on the detection rate of TB would require prospective randomized studies.

## Conclusion

Performing a sagittal image fusion in addition to the standard the axial fusion improves the accuracy to detect PCa by targeted biopsies performed with a sensor-based MRI/US fusion platform.

## Additional files

Additional file 1: Table S1. Cancer Detection Rates in Group A and B excluding men with abnormal DRE. (DOCX 19 kb)
Additional file 2: Table S2. Cancer Detection Rate and Gleason pattern in Group A and B excluding men with abnormal DRE and including only men with prior negative biopsy. (DOCX 19 kb)

## Abbreviations

DCE: Dynamic contrast-enhanced sequences
DWI: Diffusion-weighted
ESUR: European society of urogenital radiology
mpMRI: Multiparametric magnetic resonance imaging
MRI/US: MRI/ultrasound
PCa: Prostate cancer
SB: Systematic transrectal prostate biopsy
TB: MRI/ultrasound fusion guided targeted biopsy
