# Supplementary material for: The addition of a sagittal image fusion improves the prostate cancer detection in a sensor-based MRI /ultrasound fusion guided targeted biopsy
Source: BMC Urol. 2017 Jan 13;17:7. doi: 10.1186/s12894-016-0196-9 (PMC5234255; doi:10.1186/s12894-016-0196-9)
Supplement: Additional file 1: Table S1. — Cancer Detection Rates in Group A and B excluding men with abnormal DRE. (DOCX 19 kb) [file 12894_2016_196_MOESM1_ESM.docx]

SupplementalTable 1.

Cancer Detection Rates in Group A and B excluding men with abnormal DRE

|  | **Group A**  **(n=139)** | **Group B**  **(N=63)** | p-value |
| --- | --- | --- | --- |
| Overall CDR  SB  TB | 95 (68%)  86 (62%)  71 (51%) | 52 (83%)  47 (75%)  44 (70%) | 0.041  0.081  0.014 |
| *PI-RADS 3 (n=38)*  Overall CDR  SB  TB | 16 (53%)  14 (47%)  13 (43%) | 5 (63%)  3 (38%)  2 (25%) | 0.709  0.709  0.440 |
| *PI-RADS 4 (n=108)*  Overall CDR  SB  TB | 49 (65%)  42 (55%)  34 (45%) | 26 (81%)  25 (78%)  22 (69%) | 0.110  0.031  0.034 |
| *PI-RADS 5 (n=56)*  Overall CDR  SB  TB | 30 (91%)  30 (91%)  24 (73%) | 21 (91%)  19 (83%)  20 (87%) | >0.999  0.429  0.322 |
| Detected GS ≥ 7 in TB  Missed PCa  (GS ≥ 7) in TB | 54 (57%)  16 (30%*) | 38 (73%)  5 (13%*) | 0.076  0.325 |

CDR= Cancer Dection Rate; GS = Gleason Score;

SB = Random Biopsy; TB = Target biopsy; * % of GS ≥ 7 detected by TB
