# Supplementary material for: The addition of a sagittal image fusion improves the prostate cancer detection in a sensor-based MRI /ultrasound fusion guided targeted biopsy
Source: BMC Urol. 2017 Jan 13;17:7. doi: 10.1186/s12894-016-0196-9 (PMC5234255; doi:10.1186/s12894-016-0196-9)
Supplement: Additional file 2: Table S2. — Cancer Detection Rate and Gleason pattern in Group A and B excluding men with abnormal DRE and including only men with prior negative biopsy. (DOCX 19 kb) [file 12894_2016_196_MOESM2_ESM.docx]

Supplemental Table 2.

Cancer Detection Rate and Gleason pattern in Group A and B excluding men with abnormal DRE and including only men with prior negative biopsy

|  | **Group A**  **(n=96)** | **Group B**  **(N=32)** | p-value |
| --- | --- | --- | --- |
| Overall CDR  SB  TB | 64 (67%)  56 (58%)  46 (48%) | 27 (84%)  24 (75%)  23 (72%) | 0.072  0.139  0.024 |
| *PI-RADS 3 (n=24)*  Overall CDR  SB  TB | 14 (64%)  12 (54%)  11 (50%) | 1 (50%)  0 (0%)  1 (50%) | >0.999  0.478  >0.999 |
| *PI-RADS 4 (n=67)*  Overall CDR  SB  TB | 32 (60%)  26 (49%)  22 (42%) | 10 (71%)  9 (64%)  7 (50%) | 0.544  0.376  0.763 |
| *PI-RADS 5 (n=37)*  Overall CDR  SB  TB | 18 (86%)  18 (86%)  13 (62%) | 16 (100%)  15 (94%)  15 (94%) | 0.243  0.618  0.050 |
| Detected GS ≥ 7 in TB  Missed PCa  (GS ≥ 7) in TB | 31 (48%)  7 (23%*) | 18 (67%)  3 (17%*) | 0.167  >0.999 |

CDR= Cancer Dection Rate; GS = Gleason Score;

SB = Random Biopsy; TB = Target biopsy; * % of GS ≥ 7 detected by TB
